# Effect of Viewing Smoking Scenes in Motion Pictures on Subsequent Smoking Desire in Audiences in South Korea

**DOI:** 10.2196/publichealth.7093

**Published:** 2017-07-17

**Authors:** Minsung Sohn, Minsoo Jung

**Affiliations:** ^1^ Department of Public Health Sciences Graduate School of Korea University Seoul Republic Of Korea; ^2^ Department of Health Science Dongduk Women's University Seoul Republic Of Korea

**Keywords:** film, smoking, craving, South Korea

## Abstract

**Background:**

In the modern era of heightened awareness of public health, smoking scenes in movies remain relatively free from public monitoring. The effect of smoking scenes in movies on the promotion of viewers’ smoking desire remains unknown.

**Objective:**

The study aimed to explore whether exposure of adolescent smokers to images of smoking in fılms could stimulate smoking behavior.

**Methods:**

Data were derived from a national Web-based sample survey of 748 Korean high-school students. Participants aged 16-18 years were randomly assigned to watch three short video clips with or without smoking scenes. After adjusting covariates using propensity score matching, paired sample *t* test and logistic regression analyses compared the difference in smoking desire before and after exposure of participants to smoking scenes.

**Results:**

For male adolescents, cigarette craving was significantly higher in those who watched movies with smoking scenes than in the control group who did not view smoking scenes (*t*_307.96_=2.066, *P*<.05). In the experimental group, too, cigarette cravings of adolescents after viewing smoking scenes were significantly higher than they were before watching smoking scenes (*t*_161.00_=2.867, *P*<.01). After adjusting for covariates, more impulsive adolescents, particularly males, had significantly higher cigarette cravings: adjusted odds ratio (aOR) 3.40 (95% CI 1.40-8.23). However, those who actively sought health information had considerably lower cigarette cravings than those who did not engage in information-seeking: aOR 0.08 (95% CI 0.01-0.88).

**Conclusions:**

Smoking scenes in motion pictures may increase male adolescent smoking desire. Establishing a standard that restricts the frequency of smoking scenes in films and assigning a smoking-related screening grade to films is warranted.

## Introduction

Media exposure might detrimentally affect health behavior by influencing short- and long-term attitudes and beliefs [1]. In particular, movies can have a strong exposure effect. Smoking scenes that are included in the development of a plot can induce smoking behavior because they have a strong ripple effect [2-4]. Scenes of actors smoking in movies can contribute to the view that smoking is a positive, socially acceptable act [5].

Different theories have been proposed to explain how media exposure can alter health behavior. The cognitive priming theory proposes that particular stimuli provided by the media can activate related behavior in viewers [6]. The theory explains that the media can remind viewers of already acquired behavior. For example, if smokers see smoking scenes through the media, they are likely to have a stronger desire to smoke. A second theory is the theory of social learning, which posits that social people can engage in vicarious learning after witnessing reinforcements acquired through a particular behavior [7]. Learning in adolescents occurs through the indirect experience of imitating others in their social environment and the direct experience of trial and error. Mass media exposure can be a source of imitative learning. For example, a movie in which an actor smokes and receives a positive reward can instill a smoking-positive memory in viewers, which can influence future real-life behavior. A third theory is the contextual effect theory. It states that individuals may perform a particular behavior because of social contexts, not because of a desire for the behavior [8]. In other words, a particular behavior may become socially normal when presented in the media [9]. As an example, if the media portray adolescent smoking as a quotidian phenomenon, viewers are likely to have a positive context for smoking. However, the specific effects of media exposure on adolescents have not been fully studied. In particular, sensation-seeking and information-seeking behaviors on adolescents’ smoking desire as a result of media exposure are influential. Even if other conditions are equal, the desire to smoke in an adolescent who views smoking scenes in films can be stimulated or restrained significantly, depending on whether or not the person is a sensation seeker or a health-information seeker [10-12].

In 2008, the United States National Cancer Institute (NCI) reported a strong causal relationship between smoking scenes in movies and initiation of smoking by adolescents [5,13]. The Hollywood movie industry has spent millions of dollars to stage smoking scenes in movies [14]. A study on movies released in the United States from 2002-2010 chronicled that 80% of all R-rated movies and nearly 70% of all PG-13-rated movies featured smoking scenes [5,15]. Consequently, the NCI’s warning has important implications. Smoking scenes are relatively free from public health monitoring even though movies are viewed worldwide. The fact that smoking scenes can promote viewers’ smoking has not been widely disseminated [5,16,17].

In 2005, South Korea ratified the Framework Convention on Tobacco Control (FCTC) legislated by the World Health Organization (WHO) [18]. Article 13 of the FCTC recommends the inclusion of warning messages or labels in the advertisement, promotion, and support of tobacco products and restricts the use of incentives to encourage their purchase. Yet, active measures have not been instituted in South Korea. Movies rated for viewers 15 years of age or older frequently feature smoking scenes [19]. While viewing motion pictures is regarded as a popular leisure activity in contemporary culture, the presentation of smoking scenes in films and their effects on audiences have not been fully studied.

Using a large Web-based panel, we investigated whether exposure of adolescent smokers to images of smoking in movies could stimulate their smoking desire after adjusting for the factors related to smoking desire, which included their attitudes and beliefs regarding smoking, its health effects, and whether or not their parents indulged in smoking.

## Methods

### Sample

Data were derived from a survey of respondents drawn from a nationally representative Web-based sample of 748 Korean high school students (376 males and 372 females) who participated in the Hankook Research Master Sample Panel. Respondents were recruited using a dual sampling frame. A combination of random digit dial and address-based sampling allowed for the selection of a sample of individuals without using telephone land lines. Respondents received a nominal cash incentive (US $2.50) to participate when they completed both the baseline and post-movie surveys. The final response rate was 81.0%. Missing values of survey questions for key analytical variables were excluded using the pairwise method.

### Study Design

The students (aged 16-18 years) were randomly assigned to watch three professionally edited short (40-120 seconds) video clips of representative popular Korean films with smoking scenes (exposure group, n=374) or without smoking scenes (non-exposure group, n=374). Before the participants started the Web-based survey, the statistician randomly assigned each of them to two different study groups (exposure/non-exposure), according to a table of computational random numbers. The random assignment was blocked by gender to distribute males and females equally in each group. The video clips were professionally edited versions of representative hit films from among 51 films that played in Korean cinemas from 2000-2013 [19]. We defined a hit film as a movie that had been viewed by at least 5 million people [19]. The participants watched the three video clips in a row by themselves while completing the Web-based survey. The participants were unable to skip watching certain videos. In the exposure group, all scenes presented contained images of smoking. Participants’ desire to smoke was gauged immediately after viewing the clips. We examined whether smoking scenes in motion pictures could increase adolescents’ smoking desire. We also examined whether adolescents’ smoking desire could be promoted or restrained by predisposing conditions, such as sensation-seeking or health information-seeking behavior. After adjusting covariates using propensity score matching (PSM), we conducted a paired-sample *t* test to compare the smoking desire of respondents before and after viewing smoking scenes.

### Measures

Survey questions were based on previous reports on the effects of social context, including mass media use and social capital, on health [17,19,20]. The questionnaire included topics adapted from the Health Information National Trends Survey of validated measures developed through a series of health communication studies in Korea [12,21]. Sensation-seeking behavior was defined as an individual’s innate propensity to seek out novel, strong, and intricate experiences and feelings and to willingly take physical and socioeconomic risks to have such experiences and feelings [22,23]. Sensation-seeking is strongly associated with a variety of illegal and risky behaviors, such as smoking, among adolescents [24]. The Cronbach alpha values of all individual subscales ranged from .60 to .87 in this study.

#### Dependent Variable

The dependent variable of cigarette craving was measured using two questions. This was the first question: “What is your current smoking desire on a scale of 0 to 100 score?” A 0-100 Likert scale was used to evaluate responses. This was the second question: “Do you wish to take a puff on a cigarette now?” Response options to the second question were “not at all” (including non-smokers), “somewhat,” “considerably,” and “very strongly.” The first continuous question was used for *t* test analysis. The second nominal question was used for the logistic regression, with answers grouped into two categories as dependent variables by calculating the change in smoking desire before and after watching the smoking scenes using these two questions. Responses indicating an increase in smoking desire after watching the smoking scenes were coded 1 and decreased or no change in smoking desire after watching the smoking scenes was coded 0.

#### Independent Variable

We considered viewing smoking scenes in motion pictures as the baseline independent variable for the *t* test. For logistic regression, we used three independent variables (mass media, impulsivity, and information-seeking behavior) to identify related factors with difference in smoking desire before and after watching the movie clips. General mass media usage was assessed by querying how much time was estimated to have been spent each day in the prior 7 days watching television, searching for information with a smartphone, and reading news on the Internet using a personal computer. Responses ranged from 0 to ≥5 hours. Media usage was divided into four categories: ≤30 minutes, 30 minutes to 1 hour, 1-2 hours, and >2 hours (Cronbach alpha=.60). To assess the sensation-seeking behavior (ie, impulsivity) of the adolescent respondents, we used the validated and reliable Korean version of the Sensation Seeking Scale [25], which queried the responses to the following, with responses rated on a four-point Likert scale: (1) I want to try rock climbing, I want to try thrilling things even if they’re scary, I want to try thrilling sports like water skiing and surfboarding, I want to jump off an airplane with a parachute on, I want to slide down fast on skis from a high slope, I want to try bungee jumping, I want to go on roller coasters or thrilling rides at amusement parks, and I want to hang out with motorcycle gangs who look like they’re around my age (Cronbach alpha=.87); (B) I want to make out with someone of the opposite sex, I want to go to bars or clubs and have fun all night, I want to try drinking or smoking without having to mind other people, I want to laugh and be rowdy at gatherings or parties full of people, I want to go crazy at standing seats at concerts, I want to try outlandish hairstyles or clothes, I want to zoom down big roads in a sports car, and I want to sing or yell loudly in the street in the middle of the night (Cronbach alpha=.82). For information-seeking behavior, the respondents were asked “Have you ever actively searched for health-related information?” The five possible responses were as follows: “very actively,” “actively,” “average,” “inactively,” and “very inactively.”

#### Covariates

We considered covariates in accordance with previous studies [26,27]. Respondents were queried on their attitudes and beliefs with regard to smoking and its health effects. Attitude toward smoking was assessed by asking “What are your thoughts on smoking?” and responses were categorized as “very positive,” “positive,” “so-so,” “negative,” or “very negative.” Belief in the effect of smoking on health was assessed based on the question “What do you think that how much smoking will affect your health?” and responses were categorized as “no effect,” “has somewhat effect,” “considerable effect,” or “fatal effect.” Respondents were also asked “Who is a smoker among your parents?” The response options were “father only,” “mother only,” “both,” or “neither of them.” We grouped these answers into two categories: “neither of them” and “more than one.”

#### Statistical Analyses

We expected that the two groups (with exposure to smoking scenes or without such exposure) were different in a range of characteristics. Such a difference might artificially inflate the effect of smoking desire when using traditional statistical techniques [28]. PSM is a statistical tool to create matched sets of treated and untreated participants who show similar values of propensity scores (PS) for covariates [29,30]. Therefore, PSM was used in this study to compare the smoking desire of the treatment group that viewed smoking scenes with the comparison group that did not view those scenes, according to the smoking-related risk factors of attitude to smoking, belief in the effect of smoking on health, and parental smoking. PS was calculated for each gender using a non-parsimonious multiple logistic regression model to ensure that the balancing property of the covariates was satisfied. Subsequently, PS was used to match using the Mahalanobis nearest-neighbor matching algorithm without caliper. We performed a series of statistical analyses to determine the effect of viewing smoking scenes in motion pictures on audiences' subsequent smoking desire. First, as a manipulation check, chi-square tests were conducted to evenly compare the distribution of covariates between the control group and the treatment group after matching the scores. Second, the two matched patient groups were compared using a paired *t* test to identify the difference between the two groups. Third, logistic regression was performed to explore the influential factors on audiences' smoking desire.

#### Ethics Statements

Approval for the study was granted by the Korea National Institute for Bioethics Policy Institutional Review Board (Approval Date: March 8, 2016; Approval No.: P01-201603-22-003). All participants gave written informed consent to participate in this study. The Ethics Committee of the Demographic Health Survey approved the consent procedure. Any information that could distinguish individual participants was not collected during the data collection process.

## Results

### Baseline Characteristics Related to Smoking Desire

Results of a cross-tabulation analysis conducted to verify whether the experimental group and the control group were well matched are shown in Tables 1 and 2. For male respondents, the multivariate imbalance measure was 0.162 before matching and decreased to 0.111 after matching, indicating good matching. Male respondents with a “very negative” opinion of smoking comprised 60.5% of the control group and 56.8% of the experimental group. Concerning the effect of smoking on health, 67.3% of respondents in the control group and 59.3% of respondents in the experimental group believed smoking was fatal. Similar percentages of both groups responded that one or both of their parents smoked or that both were non-smokers.

For female respondents, the multivariate imbalance measure decreased from 0.246 before matching to 0.209 after matching. The proportion of females with a very negative attitude to smoking was 63.4% in both the control group and the experimental group. Similar percentages of females believed that smoking is fatal (66.0% and 68.6% in the control and experimental group, respectively). Concerning parental smoking, 54.9% of the control group responded that both parents were non-smokers with 56.2% of the experimental group responding that one or both smoked.

### Smoking Desire According to Exposure to Smoking Scenes After Propensity Score Matching

**Figure 1:**
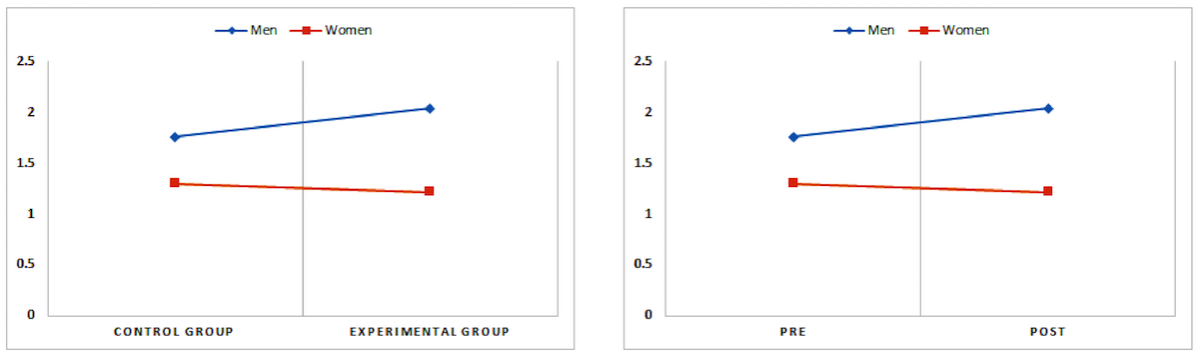
Difference in cigarette cravings between the control group and the experimental group or between before and after viewing smoking scenes in motion pictures in the experimental group.

Table 3 presents the data concerning cigarette cravings between the experimental and control groups based on independent sample *t* tests. Cigarette craving was significantly higher in the experimental group than that in the control group among male adolescents (*t*_307.96_=2.066, *P*<.05; Table 3). In the experimental group, cigarette cravings of adolescents after viewing smoking scenes were significantly higher than they were before watching the video clips (*t*_161.00_=2.867, *P*<.01; Table 4). Figure 1 depicts the results graphically.

**Table 1:** Baseline characteristics related to smoking desire (men).

Characteristics		Before matching			After matching		
		Control (n=189), n (%)	Experiment (n=187), n (%)	*p*	Control (n=162), n (%)	Experiment (n=162), n (%)	*p*
**Attitude to smoking**							
	Very positive	0 (0.0)	4 (2.2)	.04	0 (0.0)	1 (0.6)	.58
	Positive	4 (2.1)	3 (1.6)		2 (1.2)	3 (1.9)	
	So-so	28 (14.8)	38 (20.3)		21 (13.0)	29 (17.9)	
	Negative	48 (25.4)	40 (21.4)		41 (25.3)	37 (22.8)	
	Very negative	109 (57.7)	102 (54.5)		98 (60.5)	92 (56.8)	
**Belief in the effect of smoking on health**							
	Not effective	2 (1.1)	2 (1.1)	.22	2 (1.2)	2 (1.2)	.45
	Somewhat effective	19 (10.1)	17 (9.1)		11 (6.8)	17 (10.5)	
	Considerably effective	52 (27.5)	48 (25.7)		40 (24.7)	47 (29.0)	
	Fatal	116 (61.4)	120 (64.2)		109 (67.3)	96 (59.3)	
**Parental smoking**							
	Neither of them	97 (51.3)	99 (52.9)	.42	80 (49.4)	80 (49.4)	>.99
	More than one	92 (48.7)	88 (47.1)		82 (50.6)	82 (50.6)	
Multivariate Imbalance Measure		0.162			0.111		

### Effects of Mass Media Usage, Impulsivity, and Information-Seeking Behavior on Smoking Desire Among Male Adolescents

Logistic regression was performed through PSM to determine factors associated with changes in cigarette cravings among the male high school students following viewing of smoking scenes in motion pictures. Results are summarized in Tables 5 and 6. Table 5 provides descriptive statistics of the sample. According to Model III, the video clips did not significantly influence cigarette craving (Table 6). However, more impulsive adolescents had significantly higher cigarette cravings than less impulsive adolescents: adjusted Odds Ratio (aOR) 3.40 (95% CI 1.40-8.23). On the contrary, cigarette cravings did not significantly increase in adolescents who habitually engaged in health information-seeking. In particular, those who very actively sought health information had considerably lower cigarette cravings than the group who did not engage in such information-seeking: aOR 0.08 (95% CI 0.01-0.88).

**Table 2:** Baseline characteristics related to smoking desire (women).

Characteristics		Before matching						After matching					
			Control (n=185), n (%)		Experiment (n=187), n (%)		*p*		Control (n=153), n (%)		Experiment (n=153), n (%)		*p*
**Attitude to smoking**													
	Very positive		0 (0.0)		2 (1.1)		.01		0 (0.0)		0 (0.0)		>.99
	Positive		6 (3.2)		3 (1.6)				3 (2.0)		3 (2.0)		
	So-so		21 (11.4)		15 (8.0)				15 (9.8)		15 (9.8)		
	Negative		60 (32.4)		38 (20.3)				38 (24.8)		38 (24.8)		
	Very negative		98 (53.0)		129 (69.0)				97 (63.4)		97 (63.4)		
**Belief in the effect of smoking on health**													
	Not effective		0 (0.0)		0 (0.0)		.05		0 (0.0)		0 (0.0)		.23
	Somewhat effective		7 (3.8)		10 (5.3)				4 (2.6)		9 (5.9)		
	Considerably effective		59 (31.9)		40 (21.4)				48 (31.4)		39 (25.5)		
	Fatal		119 (64.3)		137 (73.3)				101 (66.0)		105 (68.6)		
**Parental smoking**													
	Neither of them		98 (53.0)		99 (52.9)		.54		84 (54.9)		67 (43.8)		.07
	More than one		87 (47.0)		88 (47.1)				69 (45.1)		86 (56.2)		
Multivariate Imbalance Measure		0.246						0.209					

**Table 3:** Smoking desire according to exposure to smoking scenes by gender after propensity score matching.

Control (exposure to smoking scenes) versus treatment group	Control		Experiment		Degrees of freedom	*t*	*p*
	Mean	SD	Mean	SD			
Men	1.58	1.76	2.04	2.19	307.96	2.066	.04
Women	1.20	1.08	1.22	1.04	304.00	0.054	.96

**Table 4:** Smoking desire according to before and after exposure to smoking scenes by gender after propensity score matching.

Before exposure to smoking in movies versus after exposure to smoking in movies	Before exposure to smoking in movies		After exposure to smoking in movies		Degrees of freedom	*t*	*p*
	Mean	SD	Mean	SD			
Men	1.76	1.86	2.04	2.19	161.00	2.867	.01
Women	1.30	1.33	1.22	1.04	152.00	1.088	.28

**Table 5:** Characteristics of mass media usage, impulsivity, and information-seeking behavior in 162 subjects.

Characteristics		n	%
**Watch television per day on average**			
	<30 minutes	89	54.94
	30 minutes-1 hour	39	24.07
	1 hour-2 hours	22	13.58
	>2 hours	12	7.41
**Search information with a smartphone**			
	<30 minutes	48	29.63
	30 minutes-1 hour	36	22.22
	1 hour-2 hours	46	28.40
	>2 hours	32	19.75
**Read news on the Internet**			
	<30 minutes	68	41.98
	30 minutes-1 hour	38	23.46
	1 hour-2 hours	33	20.37
	>2 hours	23	14.20
Impulsivity [mean, SE (range)]		2.38	0.73 (1-4)
**Information-seeking behavior**			
	Very inactively	6	3.70
	Inactively	20	12.35
	Average	92	56.79
	Actively	23	14.20
** **	Very actively	21	12.96

## Discussion

### Principal Findings

This study revealed a direct link between viewing smoking scenes and immediate subsequent smoking desire. In particular, male adolescents who watched smoking scenes were more likely to have the desire to smoke than those who watched scenes that did not contain smoking. This is consistent with findings in the literature that people 18 to 25 years of age are influenced by smoking scenes in movies and are more apt to initiate smoking [31,32]. Presently, cigarette cravings in male high-school students were markedly increased in those displaying impulsive behavior, which is a known risk factor for smoking. The urge to smoke following viewing of the smoking scenes was considerably less in those who habitually engaged in health information-seeking behavior. The former group perceived tobacco as a substance of curiosity. In contrast, the latter group had negative attitudes and beliefs regarding tobacco, which were reinforced by information-seeking.

One-third of smokers start smoking when they are adolescents. Failure to quit smoking during adolescence considerably increases the chance of becoming a long-term smoker [33]. Smoking scenes frequently appear in movies rated PG-15. This makes it very likely that Korean adolescents will view the scenes. In impressionable individuals, the exposure could be an important factor in their decision to smoke. One factor could be a reinforcement of their burgeoning positive attitude towards cigarettes. Appropriate regulations and management are necessary to curb this influence. Banning smoking scenes in films is seemingly an obvious option. However, filmmakers counter that dramatic necessity and freedom of expression can allow for depiction of smoking, especially when it is deemed essential in the storyline [34].

**Table 6:** Effects of mass media use, impulsivity, and information-seeking behavior on smoking desire of male adolescents.

Characteristics		Model 1		Model 2		Model 3	
		aOR^a^	95% CI	aOR	95% CI	aOR	95% CI
**Watch television per day on average (ref: <30 minutes)**		1		1		1	
	30 minutes-1 hour	1.64	0.52-5.17	1.45	0.43-4.94	1.13	0.34-4.50
	1 hour-2 hours	1.6	0.41-6.22	1.70	0.42-6.93	1.76	0.41-7.55
	>2 hours	2.08	0.36-11.90	1.87	0.29-11.94	1.70	0.23-12.68
**Search information with a smartphone (ref: <30 minutes)**		1		1		1	
	30 minutes-1 hour	1.46	0.32-6.64	1.63	0.34-7.71	1.64	0.33-8.21
	1 hour-2 hours	3.16	0.72-11.75	2.79	0.60-10.39	3.03	0.64-10.47
	>2 hours	3.28	0.62-15.47	2.61	0.43-12.04	2.01	0.29-10.32
**Read news on the Internet (ref: <30 minutes)**		1		1		1	
	30 minutes-1 hour	0.58	0.16-2.09	0.64	0.17-2.35	0.64	0.17-2.49
	1 hour-2 hours	0.46	0.11-1.88	0.53	0.12-2.33	0.44	0.09-2.08
	>2 hours	0.49	0.11-2.26	0.66	0.13-3.42	0.69	0.12-4.10
Impulsivity				3.13^b^	1.35-7.28	3.40^b^	1.40-8.23
**Information-seeking behavior (ref: Very inactively)**						1	
	Inactively					0.19	0.02-1.86
	Average					0.12^c^	0.02-0.92
	Actively					0.11^c^	0.01-0.89
	Very actively	** **	** **	** **	** **	0.08^c^	0.01-0.88

^a^aOR=adjusted attitude to smoking, belief in the effect of smoking on health, and parental smoking. Dependent variable: increasing smoking desire after exposing to smoking scenes (1), decreasing or no change in smoking desire after exposing to smoking scenes (reference: 0).

^b^Significance at 1% significance level.

^c^Significance at 5% significance level.

Some countries, such as the United Kingdom, have banned the promotion and advertisement of tobacco. However, they still allow movies with positive messages towards tobacco to be viewed. Many R-rated movies produced in the United States from 2001 to 2006 had smoking scenes and 90% of viewers of such movies reported that they had a greater urge to smoke after watching these movies [35,36]. However, more people agree that regulations against smoking scenes in movies are needed. Positive changes to regulate smoking scenes in movies are being made. In the United States, Hollywood film companies are practicing self-regulatory rules to ban scenes where protagonists smoke in G, PG, and PG-13 movies [37]. This decision was based on research suggesting that the more R-rated movies a minor watches, the more likely it is that this person will smoke in the future [32]. India, another country with a robust film industry (“Bollywood”) is also strengthening regulations on smoking. Tobacco brands were frequently shown in Bollywood films made in Hindi until the early 2000s [38]. However, as the government espoused the reduction of smoking scenes in movies, vetting of films to decide whether the smoking scene is necessary has been instituted [39]. To increase the effectiveness of the ban on smoking scenes, the Indian government is allowing smoking scenes but only if the scene is out-of-focus and accompanied by a 30-s message in the film warning of the danger of smoking [40].

Establishing a standard that restricts the frequency of smoking scenes in films and assigning a smoking-related screening grade to films are warranted. Our findings suggest that adolescents who attempt to quit smoking should lessen or refrain from viewing motion pictures that contain smoking scenes.

### Limitations

Some study limitations should be noted. First, the number of friends who smoke is an important covariate, but this study was not able to include any type of information on social networks among adolescents in the questionnaire due to ethical problems. However, regarding adolescent smoking, the significance of viewing smoking scenes in motion pictures may be amplified by social networks rather than attenuated [8]. Second, data on smoking desire were collected via a self-reported questionnaire. So, the results may have been subject to recall bias. In addition, the Hawthorne effect may have occurred because participants knew the purpose of the study. Future research needs to develop a longitudinal study and an investigation and management system to conclusively establish the reliability and validity of the data.

### Public Health Implications

This study reveals a direct link between viewing smoking scenes and immediate subsequent smoking desire. In particular, male adolescents who watched smoking scenes were more likely to have the desire to smoke than those who watched scenes without smoking and were more apt to commence smoking. Cigarette craving increased considerably in male high-school students with an impulsive, risk taking personality. However, the desire to smoke cigarettes was considerably decreased in those who habitually engaged in health information-seeking behavior. Thus, a comprehensive effort involving dissemination of information is needed to promote global health. Effort is especially needed to prevent adolescents from being exposed to smoking scenes in movies. First, we need to identify the more vulnerable adolescents who are being exposed to smoking scenes in films. At the same time, we need to consider a targeted approach by finding predisposing conditions, such as sensation-seeking or impulsivity. Health campaigns that foster health information-seeking among young people are a good idea. Second, rather than implementing downstream and selective protection plans targeting adolescent viewers and their parents, an upstream approach is needed. For example, if the movie industry is genuinely interested in this project, civil servants, movie creators, and academics can talk together to understand the necessity of rules against smoking scenes and find ways to come up with appropriate solutions.

## Abbreviations

aOR: adjusted odds ratio
PSM: propensity score matching
FCTC: Framework Convention on Tobacco Control
WHO: World Health Organization
